# Magma storage in a strike-slip caldera

**DOI:** 10.1038/ncomms12295

**Published:** 2016-07-22

**Authors:** J. Saxby, J. Gottsmann, K. Cashman, E. Gutiérrez

**Affiliations:** 1 School of Earth Sciences, University of Bristol, Bristol BS8 1RJ, UK; 2 Cabot Institute, University of Bristol, Bristol BS8 1UJ, UK; 3 Area de Vulcanologia, Observatorio Ambiental, MARN, San Salvador, El Salvador

## Abstract

Silicic calderas form during explosive volcanic eruptions when magma withdrawal triggers collapse along bounding faults. The nature of specific interactions between magmatism and tectonism in caldera-forming systems is, however, unclear. Regional stress patterns may control the location and geometry of magma reservoirs, which in turn may control the spatial and temporal development of faults. Here we provide new insight into strike-slip volcano-tectonic relations by analysing Bouguer gravity data from Ilopango caldera, El Salvador, which has a long history of catastrophic explosive eruptions. The observed low gravity beneath the caldera is aligned along the principal horizontal stress orientations of the El Salvador Fault Zone. Data inversion shows that the causative low-density structure extends to *ca.* 6 km depth, which we interpret as a shallow plumbing system comprising a fractured hydrothermal reservoir overlying a magmatic reservoir with 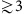 vol% exsolved vapour. Fault-controlled localization of magma constrains potential vent locations for future eruptions.

## Abstract (web summary)

The Ilopango caldera, El Salvador has had a number of large explosive eruptions, but its magmatic plumbing system is unclear. Here, Saxby *et al*. image a strike-slip faulted low-density structure under the caldera and interpret it as a gas-charged magmatic reservoir beneath a shallow hydrothermal system.

## Graphical abstract

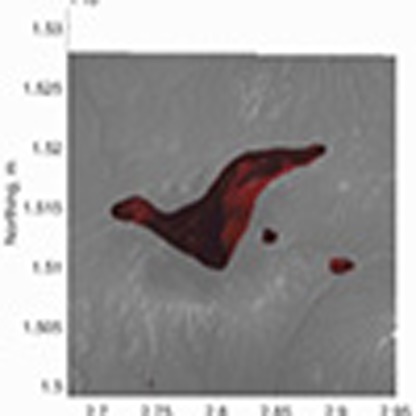

There is a well-documented association between large caldera complexes and regions of extension, including pull-apart basins in strike-slip environments (e.g., refs 1, 2, 3, 4, 5). Calderas formed in these environments tend to be both elliptical and fault-bounded. This association has led to questions regarding tectonic controls on magma accumulation, magmatic controls on strain localization, and syn-eruptive interactions between magma reservoirs and bounding faults (for example, refs 2, 6, 7, 8, 9, 10). These studies provide good evidence that the geometry of the magma reservoir controls subsidence and thus caldera geometry. The data also allow interpretations of magma accumulation controlled by both deep (pre-existing) and shallow fault structures, as well as collapse controlled by bounding faults. Unclear, however, is the extent to which regional tectonic stresses influence the geometry of magma accumulation between large caldera-forming eruptions. Addressing this question is important not only for understanding controls on the development of upper crustal magmatic systems, but also for forecasting probable locations of future eruptive activity from caldera-forming volcanoes.

The El Salvador Fault Zone (ESFZ) is a complex array of dextral strike-slip faults with a dominant E-W strike. It forms part of major tectonic lineaments in Central America (Fig. 1) that are associated with large-volume silicic calderas. The ESFZ controls the tectonics of all of central El Salvador^11^ including the area of the Ilopango caldera, an elongated 8 km by 11 km collapse structure. The morphological depression is partly filled by Lake Ilopango, which has a depth of almost 300 m. The southern caldera wall reaches up to 500 m above the lake level, giving a minimum total vertical collapse of 800 m along the caldera's southern margin. The volcano has produced at least five major eruptions of silicic tephra over the past 80,000 years, which form the explosive deposits of the Tierra Blanca eruptive sequence. The last of these, constrained to between 408 and 550 AD (ref. 12) was a VEI6+ phreatoplinian eruption which formed the widespread TBJ (Tierra Blanca Joven) deposit with a volume of >39 km^3^ Dense Rock Equivalent (D.R.E.)^13^. This catastrophic eruption disturbed the native Mayan populations as far away as eastern Guatemala^14^. The most recent volcanism in 1879–1880 formed the Islas Quemadas in the centre of Lake Ilopango and included both explosive activity and extrusion of a dacite dome that caused the lake level to rise by at least 1.2 m. The displaced water volume of 0.13 km^3^ matches the volume of the Islas Quemadas dome and suggests that pre-eruptive deformation of the caldera floor was insignificant^15^.

Here we address the role of tectonic stresses on magmatic accumulation during inter-eruptive periods between large caldera-forming eruptions with a detailed analysis of new gravity data from the Ilopango caldera. The results show that low-density material aligned along the principal horizontal stress orientations of the El Salvador Fault Zone forms a pronounced gravity low that extends to *ca.* 6 km depth beneath the caldera. The low-density structure likely maps a complex shallow plumbing system composed of magmatic and fractured hydrothermal reservoirs with a considerable vapour fraction (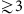 vol%). These data suggest that localized extension along the complex ESFZ controls accumulation, ascent and eruption of magma. Fault-controlled localization of magma accumulation and movement through a mechanically weak crust constrains potential vent locations for future eruptions.

## Results

### Regional Bouger anomaly

**Figure 2:**
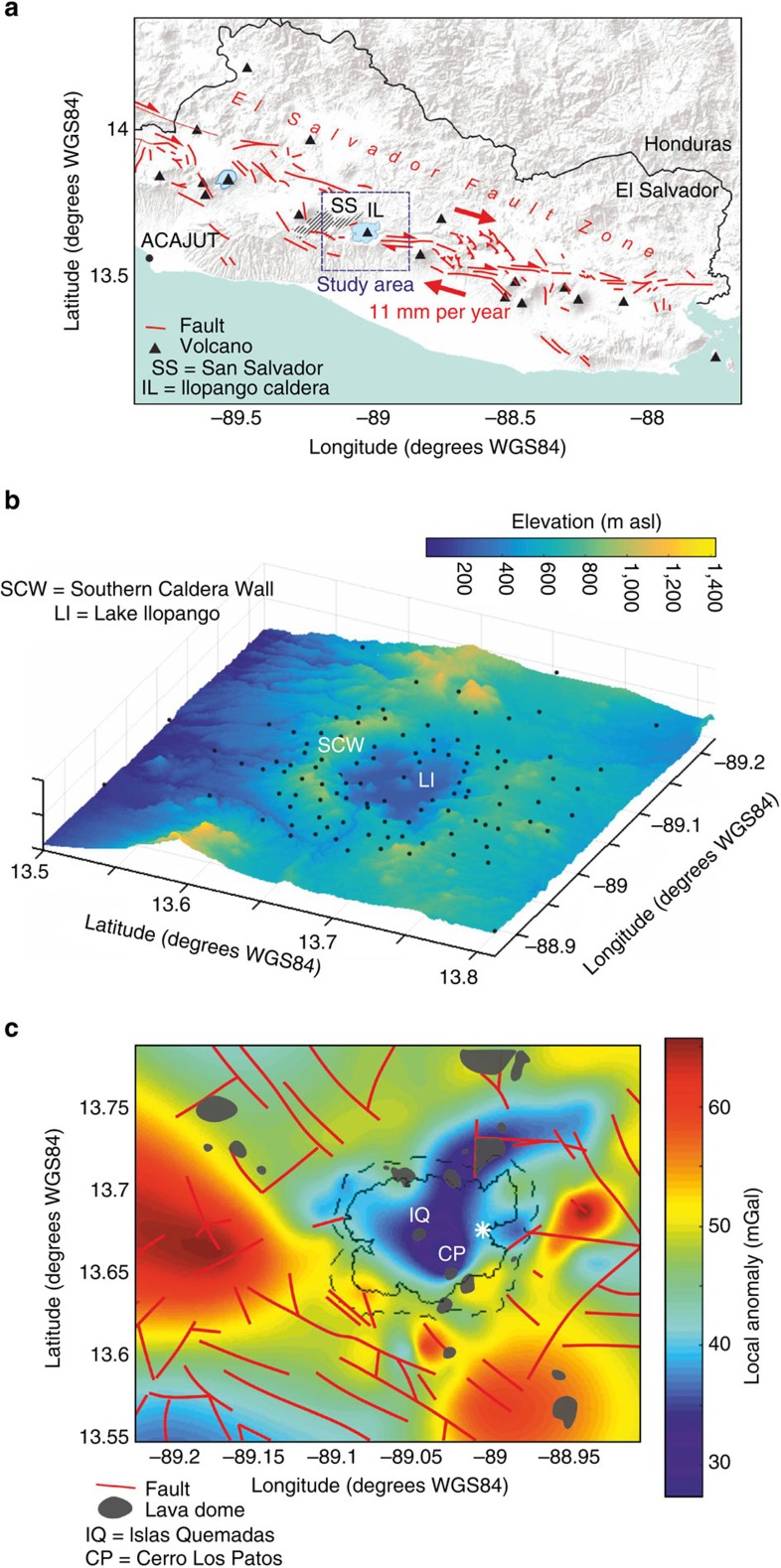
Survey area and local Bouguer anomaly maps of Ilopango caldera. (**a**) The location of the study area within the strike-slip El Salvador Fault Zone (ESFZ). ACAJUT is the absolute gravity site to which all gravity data were referenced. Red lines indicate Quaternary faults^50^,^51^,^52^,^53^. (**b**) A digital elevation model of the survey area including the benchmark locations of the joint gravimetric and GPS survey. The look direction is towards the SW. (**c**) Local Bouguer anomaly of Ilopango caldera from 106 new gravity measurements. A regional trend of 0.472 mGal/km with an azimuth of N156°E has been removed. Faults and dome structures are after^16^,^17^,^18^. Domes mentioned in text are labelled as IQ (Islas Quemadas) and CP (Cerro los Patos). Solid black line, lake outline; dashed line, inferred topographic caldera outline; asterisk, location of co-ignimbrite lag breccia.

We collected precision Bouguer gravity data in the Ilopango area immediately east of the capital city of San Salvador (Fig. 2). The >100 observation points (see Methods section and Supplementary Fig. 1 for details) include 17 measurements on the lake's shore and 4 measurements on remnant dome rocks that protrude from the lake surface and form sets of small islands. The benchmark density is necessarily low on the lake compared to its surroundings; however, the resulting Bouguer anomaly is still reasonably well constrained by data obtained on the islands and by the spacing of survey points around the lake's shore. The survey area is dominated by a distinct v-shaped gravity low with a maximum amplitude of ∼−15 mGal located within the main caldera depression close to Islas Quemadas. Peripheral gravity highs along the south and southwest of the survey area coincide with a series of mapped dome complexes (Fig. 2).

### Local Bouguer anomaly

**Figure 3:**
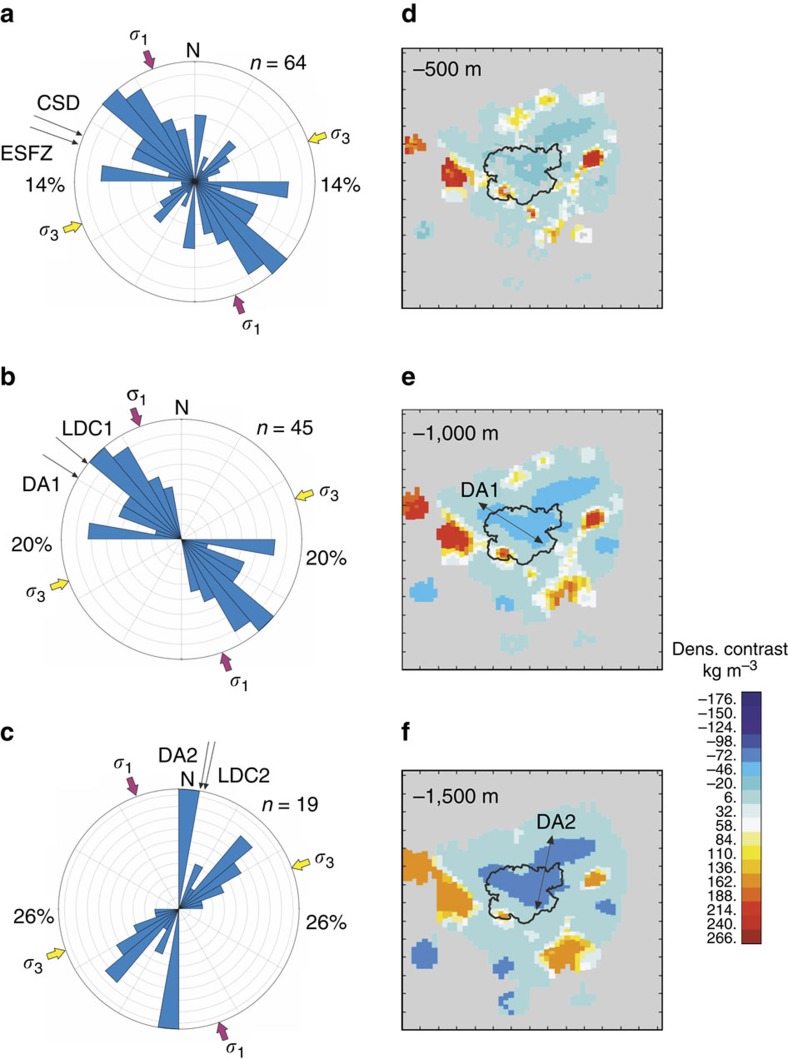
Rose diagrams and horizontal cross sections of inverse model. (**a**–**c**) Rose diagrams of local fault strikes. Arrows indicate trends of other proximal structural and density features as well as approximate principal horizontal compressive stress orientations after^11^, obtained to the East of Ilopango caldera. (**a**) All faults^16^,^17^. (**b**) NW-SE trending faults. (**c**) Extraction of N-S and NE-SW trending faults to examine these fault sets in detail. (**d**–**f**) Horizontal cross sections through the adjusted ±100 kg m^−3^ subsurface density model at depths *z*=−500 (**d**) *z*=−1,000 (**e**) and *z*=−1,500 m (**f**), respectively. The outline of Lake Ilopango is shown for reference. Spacing of tick marks on all axes is 3,000 m. CSD, strike of Cocos subduction zone; DA1, NW-SE intra-caldera limb of density anomaly; DA2=NNE-SSW limb of density anomaly; ESFZ, strike of El Salvador Fault Zone; LDC1, orientation of main lava dome chain; LDC2, orientation of north-south lava dome chain.

The main local negative Bouguer anomaly is located beneath the centre of Lake Ilopango and appears to be segmented into two limbs aligned roughly NW-SE and NNE-SSW, respectively (Figs 2 and 3). The NNE limb aligns with several post-caldera domes along and beyond the northern and north-eastern lake shore. The NW limb aligns with a broad chain of domes extending NW-SE across the survey area. The data identify the centre of the lake as the main local gravity low and the point of intersection of the two limbs.

### Inversion

**Figure 4:**
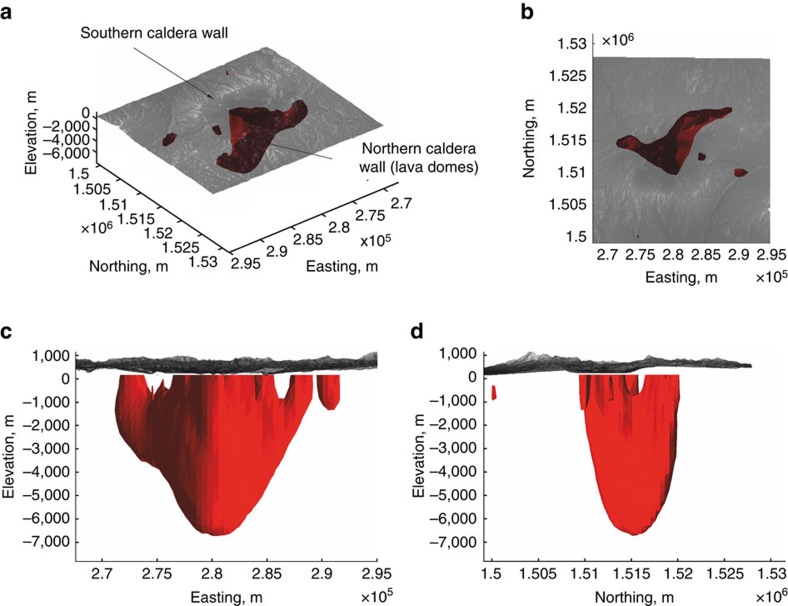
3D view of the negative density anomaly beneath the Ilopango caldera. **a** Oblique view, **b** plan view, **c** facing north, **d** facing west. The isosurface delineates a negative density contrast of −100 kg m^−3^. Minimum and maximum *a priori* density contrasts used were ±200 kg m^−3^ and the background density stratification is linear with an increase of 50 kg m^−3^ per kilometre depth. The low extends to around 6,000 m below sea level and consists of a bifurcated structure with limbs branching toward the shallow subsurface. Latitude and longitude values are in UTM.

**Figure 5:**
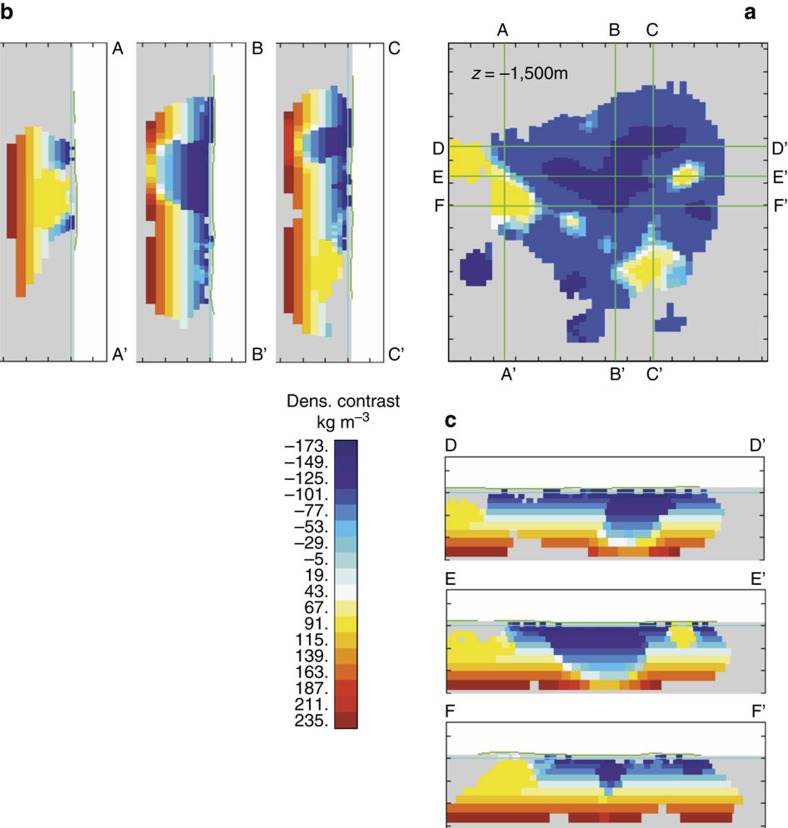
Vertical and horizontal cross sections. A horizontal (**a**) three N-S vertical sections (**b**) and three E-W vertical sections (**c**) through the subsurface density model at Ilopango. Minimum and maximum *a priori* density contrasts used were ±100 kg m^−3^. The background density stratification (shown) is linear with an increase of 50 kg m^−3^ per kilometre depth. Tick marks on all axes=3 km.

We carried out an inversion of the local gravity data to obtain a density distribution model beneath the caldera, and to constrain the vertical extent of the central anomaly (see Methods section for details on inversion routine). The central modelled negative density anomaly is a 5–6 km deep, bifurcated structure with limbs branching towards the shallow subsurface (Fig. 4). The anomaly is horizontally elongated along the trends shown by the geometry of the negative Bouguer anomaly to the NNE and NW of the lake. In addition, small high-density anomalies are modelled to the southwest of the caldera (Fig. 5).

Despite variations in the geometry of the modelled density distribution for different boundary conditions (that is, permissible density contrasts; Supplementary Figs 4 and 5), the inversion models depict a coherent, persistent and both vertically and horizontally elongated low-density body beneath the caldera. A three-dimensional visual animation of the body of negative density contrast is available in form of Supplementary Movie 1.

## Discussion

The Ilopango caldera is nested within a complex assemblage of faults of the ESFZ (Figs 2 and 3). A quantitative evaluation of local faults digitized from published maps^16^,^17^,^18^ confirms matching orientations between the mean strikes of the faults, the NW limb of the Bouguer gravity anomaly and the chain of post-caldera domes including the Islas Quemadas and Cerro los Patos (Fig. 2). There are also a significant number of faults in the survey area which strike approximately N-S, aligning with the NNE limb of the density anomaly as well as with a line of lava domes on the lake's northeast shore.

Although the possible extent of the strike-slip fault system of the ESFZ within the caldera is covered by the lake, fault slip data are available east of the caldera^11^, along the segment of the fault that ruptured during a M_*w*_ 6.7 earthquake on 13 February 2001. These data indicate a dominant transcurrent stress field along the fault, with horizontal compressive stress tensors *σ*1 and *σ*3 oriented ∼N160°E and ∼N70°E, respectively (11; Fig. 3). The NW-SE limb of the low-density structure is aligned roughly along the maximum principal compressive stress. Statistical analysis of the strike of individual faults highlights a close relationship between the tectonic lineaments and the geometry of the gravity low (Fig. 3). It thus appears that the mass deficit responsible for the local gravity low is intimately related to the existing fault systems, and the governing extensional stress field.

Bouguer gravity lows at calderas are generally interpreted as low-density infill of pyroclastic deposits from previous caldera-forming eruptions^19^. Although the last caldera-forming eruption produced the very low-density, pumice-rich^17^ Tierra Blanca sequence, the depth of the anomaly (∼6 km) and its modelled shape (Fig. 4) preclude caldera infill as the sole cause of the mass deficiency. Other possible contributors to the density low include low-density breccia and clays in fault cores, fractured host rocks in fault damage zones^20^, hydrothermal^21^ and magmatic^22^ reservoirs.

Below, we explore these contributors to the Bouguer anomaly and resultant inverse models.

One plausible interpretation of the gravity data is that the NW-SE striking limb that follows horizontal *σ*1 is associated with extensional fractures that connect two segments of the main strike-slip fault, while the NNE-SSW striking limb maps an area of block rotation. Damage zones associated with the main strike-slip fault cutting through the caldera may be an important contributor to the gravity low. Damage zones can be created by the interaction of strike-slip fault segments^23^. Such zones often show more complex and intense fracturing than damage zones formed along a single fault.

Either type of damage zone, however, can concentrate extensional stresses along synthetic and antithetic conjugated secondary faults, pull-apart fractures or block rotation^23^. Fault cores in particular, but also damage zones with a high fracture frequency or fractures that are filled with gas or low-density fluids, create permeable^24^ fracture networks and fluid pathways that degrade the mechanical competence of rock and reduce its bulk density.

Hydrothermal fluids that follow pre-existing zones of weakness in the shallow crust could hence be important contributors to the low-density signal. In fact, shallow-seated fault damage zones often connect to hydrothermal reservoirs^25^. Like many calderas worldwide^21^,^26^, Ilopango has an active hydrothermal system that manifests as chemical tracers in the lake emitted from subaqueous seeps on the lake bed^27^. Water samples show that the southern part of the lake is enriched in As, Cl^−^, B, 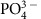 and 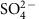, perhaps reflecting leaching of lake sediments by hydrothermal gases. Divers have reported seeps of hot water in the south of the lake near the Cerro Los Patos island (Fig. 2; (ref. 28)). The area between the Islas Quemadas and the lake's southeast margin also experienced the most frequent seismicity of any lake sector between 1984 and 2002 (ref. 27). It is likely therefore, that the hydrothermal system is located beneath the southern part of the lake; its spatial extent and geometry have not been established, but we can use abundant observations from other calderas to infer its presence in the first few kilometres beneath the caldera floor^21^.

**Figure 6:**
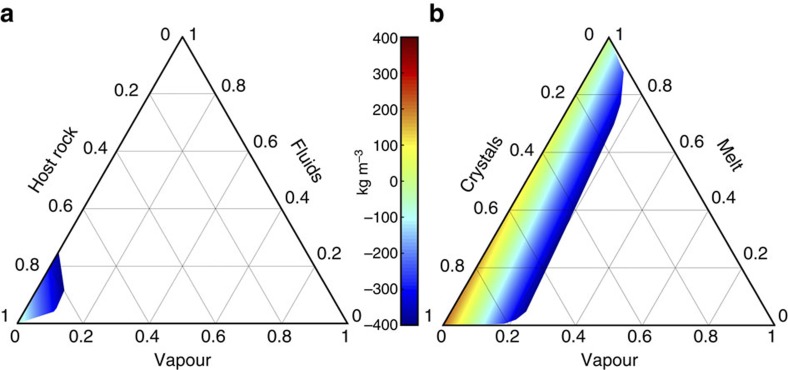
Multi-phase diagrams. Ternary multi-phase density contrast parameter space of scenario (1), a hydrothermal reservoir to explain the upper part of the modelled central negative density anomaly at ∼1 km beneath Ilopango (**a**) and scenario (2), a magma reservoir to explain the lower part of the modelled central negative density anomaly at ∼5 km beneath Ilopango (**b**). The background host rock density for (1) is calculated at 2,350 kg m^−3^ and for (2) at 2,450 kg m^−3^. Average boundary densities of the ternary phase diagrams for crystals, host rock, melt, fluids and vapour are: 2,670, 2,350, 2,450, 1,000, and 1.5 kg m^−3^, respectively. The colour bars show density contrasts in kg m^−3^. Contrasts <−400 kg m^−3^ are deemed unrealistic and are not shown. To explain a negative density contrast, a wide range of phase fraction combinations are mathematically possible including >20 vol% vapour for scenario (1) and >80 vol% melt for scenario (2), but few are geologically plausible. However, a non-zero volume fraction of vapour needs to be invoked to explain either scenario.

We favour a model whereby the upper part (≤3 km depth) of the negative density anomaly derives from an intricate combination of fractured crustal rocks that host a hydrothermal reservoir containing low-density aqueous fluids and gas. To explain the observed gravity low and the modelled anomalous low-density body one needs to invoke a 5–15% reduction in the bulk rock density. We test this by demonstrating that various permutations of major surface lithologies, including ignimbrites and lava flows with average bulk density similar to the terrain density (2,200 kg m^−3^), fractures and voids filled with hydrothermal fluids (∼1,000 kg m^−3^), and/or gas (∼1.5 kg m^−3^), can in fact account for the shallow density distribution model (Fig. 6).

To explain the negative density contrast between ∼3 and 6 km depth we test for the presence of partial melt, or a cooling, remnant silicic intrusion, or a combination of both. Remnant magma from the last (late 19th century) dome forming eruption is likely to reside beneath Ilopango. However, the density of rhyolite and dacite melt exceeds the mean terrain density, thus a large remnant pool of melt would create either a neutral or a slightly positive gravity anomaly. The same condition applies for solidified subvolcanic bodies of dacite or rhyolite (2,400–2,600 kg m^−3^). A more disconcerting possibility for hazard implications is that the gravity low records a combination of residual melt and excess gas in a partially crystallized reservoir. In fact, substantial volumes of excess gas are required to explain the density contrast from a background density of 2,450 kg m^−3^ at 5 km depth. A reasonable conservative estimate for a −100 kg m^−3^ density contrast would involve as much as 10% excess gas in a magmatic mush with 20 vol% melt. The possible presence of excess magmatic gas beneath Ilopango has implications for current hazard assessment given the absence of significant fumarolic or diffuse degassing^27^ through the caldera floor. A closed system hosting a buoyant dacite magma reservoir could respond rapidly to decompression, as documented in the VEI6 Pinatubo eruption in 1991 (ref. 29) that is often used as an analogue of the TBJ eruption. Petrological work^30^,^31^ indicates that dacitic magma can reside and evolve at shallow crustal levels at confining pressures of <100 MPa. Given the >80,000 year lifespan of a silicic magmatic system beneath Ilopango caldera and its recent dome forming activity^32^, it is plausible that the lower part of the large negative density anomaly beneath the centre of Lake Ilopango records the upper portions of an evolved silicic magma reservoir.

By contrast, the isolated chain of positive density contrasts that we model to the southwest of the central caldera depression extend from the shallow subsurface to a maximum of 5 km depth (Fig. 5). We interpret these anomalies as dense, remnant magma bodies (likely sheeted dykes), composed of fully crystallized chemically intermediate to evolved (andesitic, dacitic and rhyolitic) subvolcanic rocks. Their anomalous gravity signal and density contrast is similar to that of the dense cores of extinct dome complexes on Montserrat^33^, which have a modelled density of up to 2,600 kg m^−3^. The parallel alignment of positive density contrasts with the domes, the NW segment of the main intra-caldera gravity low, and the principal compressive stress orientation, may suggest a progressive north-eastward evolution of magma storage and extraction over time. The concentration of post-caldera lava domes across the entire horizontal extent of the main density low (Fig. 2) points towards a subsurface connection between a central magma reservoir and surface vents, including the latest eruptive vent of the Islas Quemadas. Our data suggest that multiple subsurface magma reservoirs aligned laterally along the conjugate NW-SE and NNE-SSW lineaments could have supplied magma to these post-caldera domes and are a likely source for future eruptive activity.

**Figure 7:**
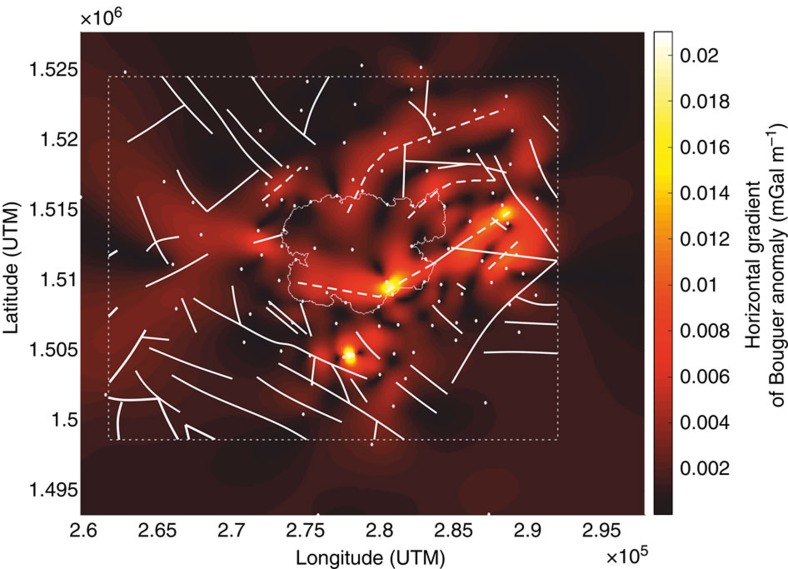
Map of horizontal gravity gradients of the survey area. Faults interpreted from the gradients are highlighted by dashed lines; faults after refs 16, 17 are shown by solid lines. Gradients are given as mGal m^−1^. The benchmark locations are shown for reference.

The derived density contrast model (Fig. 4) has significant implications for the relationship between tectonics and magmatism at Ilopango. A collapse caldera is formed by roof collapse along bounding faults into an emptying reservoir by either explosive eruption of magma or lateral withdrawal of magma^6^,^34^. To create explosive ash-flow calderas such as Ilopango, a dyke connecting a magma reservoir with the ground surface to channels the flow of magma during the eruption. The location, inclination and geometry of the dyke is controlled by the governing stress field; a dyke may either form a vertical or inclined sheet or invade a ring-like fault^6^,^7^. Calderas formed by explosive eruptions from dyke intrusion along ring faults can be geophysically characterized by arcuate and short-wavelength high-density and high-resistivity anomalies at the periphery of the caldera depression^35^,^36^. The absence of a ring-like gravimetric high at Ilopango, together with the derived subsurface density distribution, indicates that caldera collapse may not have been controlled by ring-dyke invasion along a bounding fault. Magnitudes of horizontal gradients (HG; see Methods section) of the local Bouguer anomaly are highest at the south-eastern and south-western portions of the caldera (Fig. 7). These gradients appear to map two linear subsurface structures that strike ∼N100°E and ∼N70°E. We interpret these lineaments as bounding faults of one or more caldera collapses. The lineaments are located within the current caldera depression and roughly mark Lake Ilopango's southern shore line, where the caldera wall and by inference the amplitude of vertical collapse, are highest. We therefore propose a trapdoor-like collapse of the caldera with its hinge zone located along the northern sector and its largest subsidence in the south. We cannot comment on the number of vertical collapses, but given that there have been at least five major eruptions of silicic tephra over the past 80,000 years, incremental growth of the caldera appears likely. Asymmetric (trapdoor-like) collapse is common in analogue models of caldera collapse in strike-slip tectonic regimes^2^.

**Figure 8:**
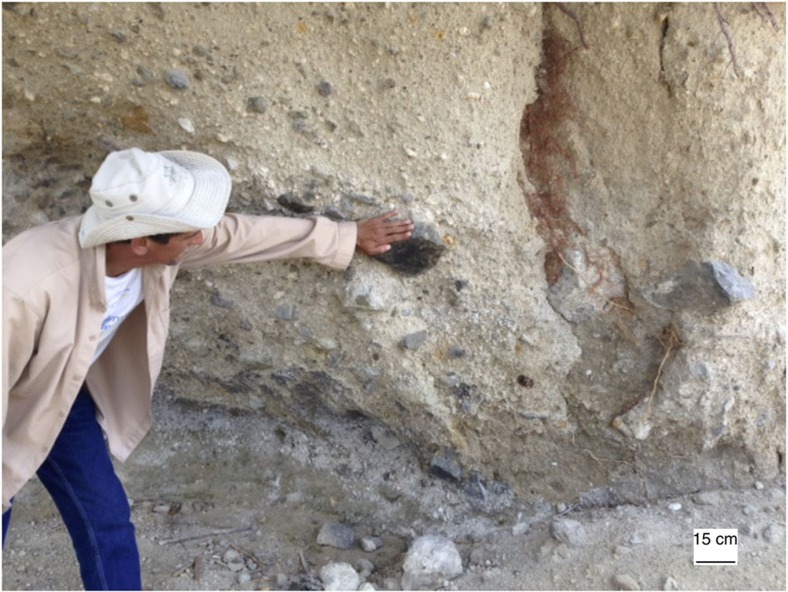
Image of a coarse and lithic-rich pyroclastic deposit interpreted to be a co-ignimbrite lag breccia. The scale bar is 15 cm. This depositional facies indicates proximity to an eruptive vent which may have been associated with the SE bounding fault of the caldera shown in Fig. 7. The outcrop is located at eastern shore of lake Ilopango (13.67304°N and 89.01202°W.)

The subsurface gravimetric image of Ilopango indicates that regional strike-slip faults can be exploited for magma accumulation and eruptive ascent, corroborating results presented by ref. 2. Furthermore, the bounding faults imaged by the HG may represent faults that were linked with, and tangential to, the margin of the magma reservoir(s) that promoted the explosive activity and caldera collapse(s). Such faults can accommodate collapse-related extension at the periphery of the caldera and may act as sites for caldera-forming eruptions^2^. A co-ignimbrite lag breccia from a large explosive eruption outcrops on the eastern lake shore and indicates proximity to an eruptive vent (Fig. 8). This deposit may be derived from an eruption centre along the SE bounding fault, but more field evidence is needed to link the explosive deposits to fault-controlled eruption centres. Faults proposed from mapped horizontal gravity gradients outside the caldera depression are dominantly aligned along ENE-WSW directions and hence match the orientation of faults along the wider ESFZ as shown in Fig. 3. Significant extensional strain rates of 3.5 × 10^−15^ s^−1^ to 4.4 × 10^−14^ s^−1^, have been constrained from proximal Tierra Blanca tuffs to an interval of quiescence at Ilopango volcano between 24 and 75 yr before present (B.P.)^37^. Our interpretation is that Ilopango caldera was primarily formed by trapdoor collapse during pull-apart basin formation and block rotation along the main strike-slip tectonics of the ESFZ, during which the significant inter-eruptive extensional strains were accommodated.

Although it may be tempting to adopt the current geophysical structure as a mirror image of the structure causative for the collapse, it is important to note that the presented image of the subsurface architecture of Ilopango caldera is not a representation of the architecture before the formation of the caldera^34^. If the lower part of the central density low maps a remnant magma body, its linear nature (Fig. 4) and alignment with the prevailing strike-slip faults (Fig. 3) suggest that local and regional stress fields currently control the accumulation and ascent of magma in this region. In this model, post-caldera eruptions at Ilopango are fed by faults that act as preferred pathways for magma ascent and shallow hydrothermal fluid circulation. Future intra-caldera eruptions at Ilopango could be induced by dyking along a strike of around N130°E (approximately horizontal *σ*_1_) or N20°E either within the caldera beneath the lake or within a few kilometres of its northern shore. It is also possible that a major faulting event at the caldera could induce volcanic activity. Either scenario poses a risk to the population of San Salvador.

## Methods

### Gravity survey and data processing

Joint gravity and positioning data were collected from 106 benchmarks around Ilopango caldera in March 2015. Points within 1 km of the lake shore, and on remnant dome rocks within the lake, have an average spacing of 1.4 km (minimum 0.9 km, maximum 2.6 km), with a sparser coverage in distal areas (Supplementary Fig. 1). Benchmark positioning data were collected using a TOPCON HiPer Pro GNSS base and rover system with the rover recording for 8–20 min at 0.5 Hz. Gravity data were obtained using a Scintrex CG-5 Autograv gravimeter (#572). The precision of benchmark location was generally under 0.06 m in the *z* axis and better than 0.05 m in the *x* and *y* axes after baseline processing of the base data against three IGS cGPS stations (BOGT, SSIA, MANA). Survey measurements were initially tied to a GPS and gravity reference in downtown San Salvador by occupying reference and control sites up to 6 times per day (depending on the design of measurement loops) to check for instrument drift and tares. All measurements were finally referenced to the absolute gravity site ACAJUT (Fig. 2). We obtained an average precision of 0.013 mGal (1 mGal=10^−5^ m s^−2^) for the raw gravity data, which results in 0.016 mGal after propagating elevation uncertainties. Gravity data reduction for solid Earth and ocean tidal components were performed using the Wahr-Dehant^38^ and GOT99.2 (ref. 39) latitude-dependent models, respectively, and the TSOFT package^40^. Reductions for free-air, Bouguer slab and latitude effects followed standard procedures^41^.

### Terrain correction and regional detrending

Terrain corrections are usually carried out as far as 1.5 degrees of latitude (166.735 km), matching the standard radial distance used for spherical cap formulation Bouguer corrections^42^. For this survey, which does not use a spherical cap model, a greater maximum radial correction distance of 300 km was chosen due to a significant on- and off-shore topography (Supplementary Fig. 2). This was based on the optimum correction distance determined for a Taiwan gravity survey in a location surrounded by seafloor topography of similar depth and ruggedness to that of offshore El Salvador^43^. With a correction distance this great, the uncertainties induced from nearby topographic undulations will exceed the uncertainties induced by more distant topographic features^42^.

Digital elevation model (DEM)-based techniques tend to omit the effects of large topographic variations near the gravimeter, which can reach several tens of mGal depending on the terrain and the resolution of the DEM^44^; this effect was minimized by choosing survey locations according to the lowest possible near-field topography, and noting any significant near-field topography during the survey. When conducting a gravity survey near lakes and seabed features, it is not sufficient to carry out a standard terrestrial terrain correction. Where the seafloor is at great depth, such as the slope between coastal El Salvador and the Middle America Trench, it is also important to take the gravitational attraction of the water into account^42^,^43^. This is particularly important at Ilopango as the survey surrounds a large freshwater lake. The density of water is much lower than the density of rock, and can be fixed as a known value. Therefore, by calculating the bathymetric corrections separately, the terrain density can be changed experimentally while water density is kept constant^42^. For this reason, and to increase computational efficiency, separate DEMs were built for the lake bed and seabed. In the terrestrial section, the distal elevation data creates the most computational expense^43^; to reduce this, the terrestrial DEM was split into two parts, with a higher-resolution section for proximal data and a lower-resolution section for distal data (see Supplementary Fig. 2).

The proximal terrestrial DEM was built from shuttle radar topography mission data with 90 m lateral resolution and a vertical accuracy of 16 m. The distal DEM was built from 120 m shuttle radar topography mission data, and the seafloor topography was built from a variable resolution global multi-resolution topography data set. There was no existing digital data set for the bathymetry of Lake Ilopango. For this reason, a bathymetric chart was digitized by creating a triangulated irregular network from *ca.* 500 published spot depths. The triangulated irregular network method was chosen to allow higher resolution in areas of greatest structural complexity, such as islands in the centre of the lake, while ensuring the absence of artefacts which may result from more complex methods of interpolation. The spatial resolution of the lake bathymetry ranges from tens of metres to around 0.5 km in deeper areas.

The correction was carried out using an automated script following the approach described by^45^, except that a terrain correction was obtained for each DEM grid point rather than for larger compartments. The corrections for the seafloor topography and lake bathymetry were carried out by replacing the water with rock, and then subtracting the gravitational attraction caused by the water body^43^. The terrain correction for the seawater was carried out using average seawater density (1,028 kg m^−3^) subtracted from terrain density, and the correction for the lake bathymetry used the standard density of freshwater (1,000 kg m^−3^) subtracted from terrain density.

A large wavelength regional trend of 0.472 mGal km^−1^ with an azimuth of N156° E was removed by a binomial fit to the resultant Bouguer anomaly data to derive the local Bouguer anomaly map (Fig. 2).

### Terrain density

Gravity data processing aims to model patterns of subsurface anomalous mass; therefore, corrections for the interference caused by surrounding terrain requires accurate knowledge of its density. Density is a required input for two of the corrections used in this survey: the Bouguer correction and the terrain correction, which account for the mass and position of rock around and below the survey area.

The most accurate value for average terrain density can be determined mathematically by decoupling the gravity effect due to terrain from the residual gravity anomaly. The terrain density chosen must yield as little residual correlation between gravity and elevation as possible. However, due to the nature of collapse calderas, it may be expected that gravity and topography patterns are both related to the shallow subsurface structure on wavelengths matching the size of the caldera (for example, Las Cañadas caldera, Tenerife; ref. 36). Therefore, the terrain density of choice is one which produces the least correlation between gravity and elevation at intermediate-wavelengths (between 1–5 km) among a range of density values.

As an upper bound, we first employ a standard value of 2,670 kg m^−3^, which represents the average density of the shallow (<4 km depth) silicic continental crust,^46^. The actual density was expected to be lower than average due to the abundance of low-density, pumice-rich ignimbrite deposits surrounding Ilopango; therefore, further tests were carried out using densities of 2,300, 2,000 and 1,700 kg m^−3^, respectively (Supplementary Fig. 3). To assess the remaining correlation between gravity and elevation after applying terrain corrections using these values, four cross sections across the survey area were chosen for their high density of gravity data points and significant topographic undulations (Supplementary Fig. 3). For these, the resultant Bouguer anomaly for the four different correction densities was plotted against elevation. Long-wavelength patterns are independent of correction density, suggesting that long-wavelength regional trends are present in the area. The gravity low in the caldera depression is also a ubiquitous feature, and indicates a relationship between surface and subsurface structure that is common at calderas (for example, ref. 36). The ‘correct' density can be determined from intermediate-wavelength patterns: for example, at the southern caldera wall in sections A-A′ and B-B′, and at the northern caldera wall in A-A′, a density of 1,700 kg m^−3^ produces a correlation between Bouguer anomaly and topography while a density of 2,670 kg m^−3^ produces an anti-correlation. The value with the least correlation was determined to be between 2,000 and 2,300 kg m^−3^. Further refined tests using a value of 2,200 kg m^−3^ determined that this density produced the least correlation between intermediate-wavelengths of between 1–5 km gravity and topographic features (Supplementary Fig. 3), and so 2,200 kg m^−3^ was chosen for the final terrain correction.

### Data inversion

The local Bouguer anomaly data were inverted for subsurface density contrasts by filling a subsurface grid of three-dimensional parallelepiped cells with a defined range of positive and negative density contrasts using the GROWTH2.0 inversion software^47^. Details of the inversion procedure can be found in^36^,^47^. Before inversion the subsurface volume was divided into 9441 parallelepiped cells with a minimum side length of 503 m, increasing to a maximum of 1,961 m in deeper and peripheral (lower sensitivity) zones. The cell dimensions were selected to create a balance between high model resolution and computational efficiency, with the maximum length kept smaller than the average spacing between gravity points (∼2 km). The resulting grid cells were initially filled with *a priori* density contrasts in the range of 400 to −400 kg m^−3^. A stratified background density increase of +50 kg m^−3^ km^−1^ was selected. Different *a priori* density contrasts were refined using systematic trial-and-error mathematical exploration of the model space (Supplementary Figs. 4 and 5). The reader is referred to an inversion sensitivity test presented in^36^ for a survey with a similar ratio of number of benchmarks per unit survey area to explore details on the limitation of the inversion approach and resultant models. In summary, the methodology tends to produce models involving a minimum total anomalous mass; as a result, subsurface density contrasts are likely to be stronger in reality than modelled.

### Horizontal gradient method

We derive horizontal gradients (HG) of the local Bouguer anomaly^48^,^49^ to locate the horizontal boundaries of regions of contrasting density. If G(*x*,*y*) is the local Bouguer anomaly, then the magnitude of the horizontal gradient HG(*x*,*y*) is given by ref. 49.


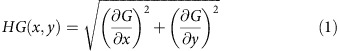


High horizontal gradients of a gravity anomaly tend to overlie the edges of tabular bodies if they are vertical and well separated from each other. The method is one of the simplest used to estimate horizontal locations of lithological contacts as it is the least susceptible to data noise^49^ and well suited to delineate sharp lithological boundaries along faults in our data set.

### Data availability

The data that support the findings of this study are available from the corresponding author on request.

## Additional information

**How to cite this article:** Saxby, J. *et al*. Magma storage in a strike-slip caldera. *Nat. Commun.* 7:12295 doi: 10.1038/ncomms12295 (2016).

## Supplementary Material

Supplementary Figures 1-5 Supplementary Figures 1-5

Supplementary Movie 1 3D animation of the central negative density anomaly beneath Ilopango caldera. The anomaly maps the -100 kg m-3 isosurface for an a priori minimum and maximum density contrasts of ±200 kg m-3 . The animation starts a view from the north with a view direction upwards towards the ground surface before the anomaly is rotated anti-clockwise by 180 degrees. The animation stops with a plan view from above.

## Figures and Tables

**Figure 1:**
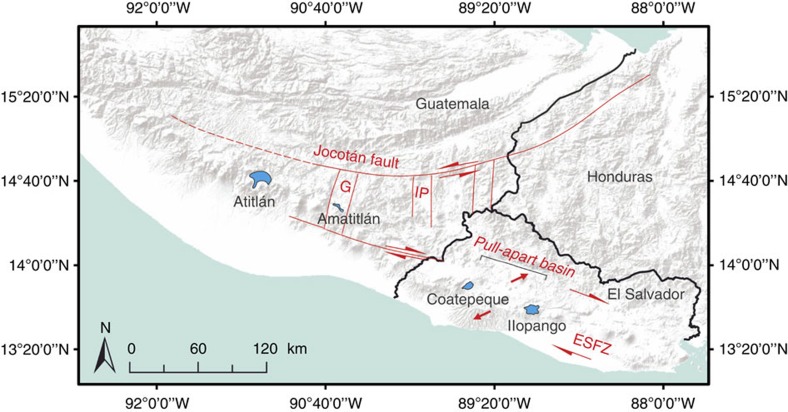
Tectonic setting of large-volume silicic calderas in El Salvador and Guatemala. The Jocotán fault is the southernmost plate boundary fault between the North American and Caribbean plates, and is the northern boundary of along-arc transtensional deformation. Modified after ref. 50. ESFZ=El Salvador Fault Zone; G, Guatemala graben; IP, Ipala graben.
