# Supplementary material for: Magma storage in a strike-slip caldera
Source: Nat Commun. 2016 Jul 22;7:12295. doi: 10.1038/ncomms12295 (PMC4961867; doi:10.1038/ncomms12295)
Supplement: Supplementary Figures 1-5 [file ncomms12295-s1.pdf]

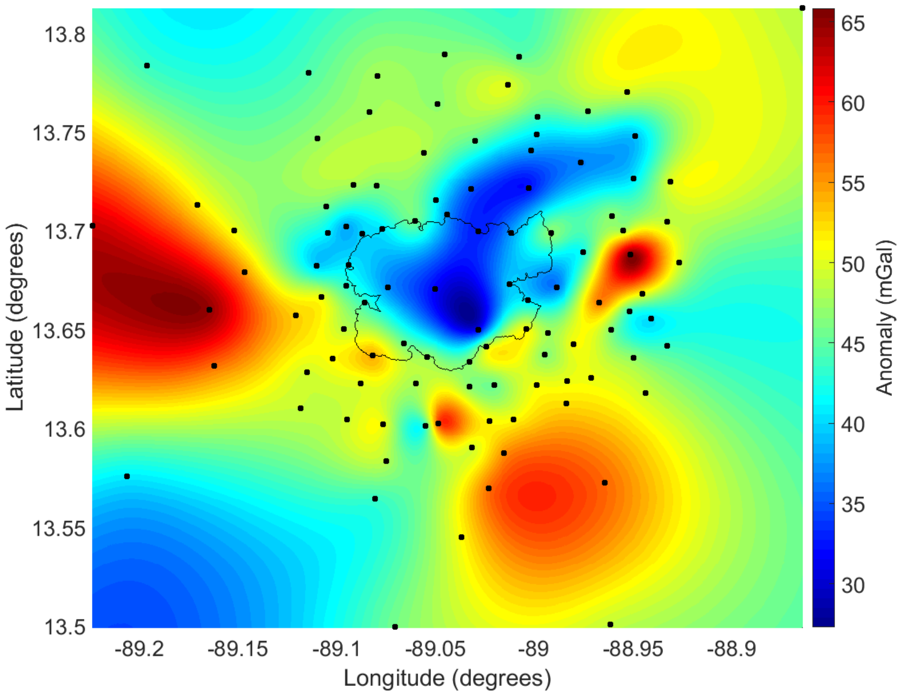

Supplementary Figure 1: Resultant regional Bouguer anomaly around Ilopango caldera from 106 new gravity stations (open circles). The anomaly has been constrained using a terrain density of 2200 kg m^{-3}.

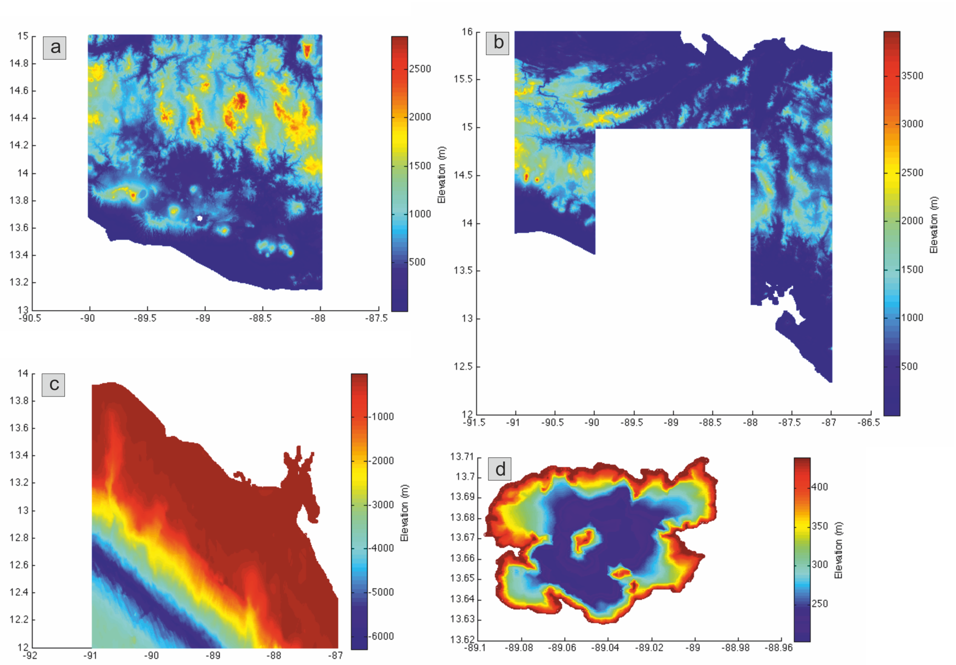

Supplementary Figure 2: Four DEMs used for terrain correction. a) proximal terrestrial SRTM data (horizontal resolution 90 m), b) distal terrestrial SRTM data (horizontal resolution 120 m), c) offshore ocean bathymetry data showing part of the Middle America Trench (variable resolution), d) bathymetry data for Lago Ilopango, built from point data taken from published bathymetry charts (variable resolution). Axes show latitude and longitude in degrees.

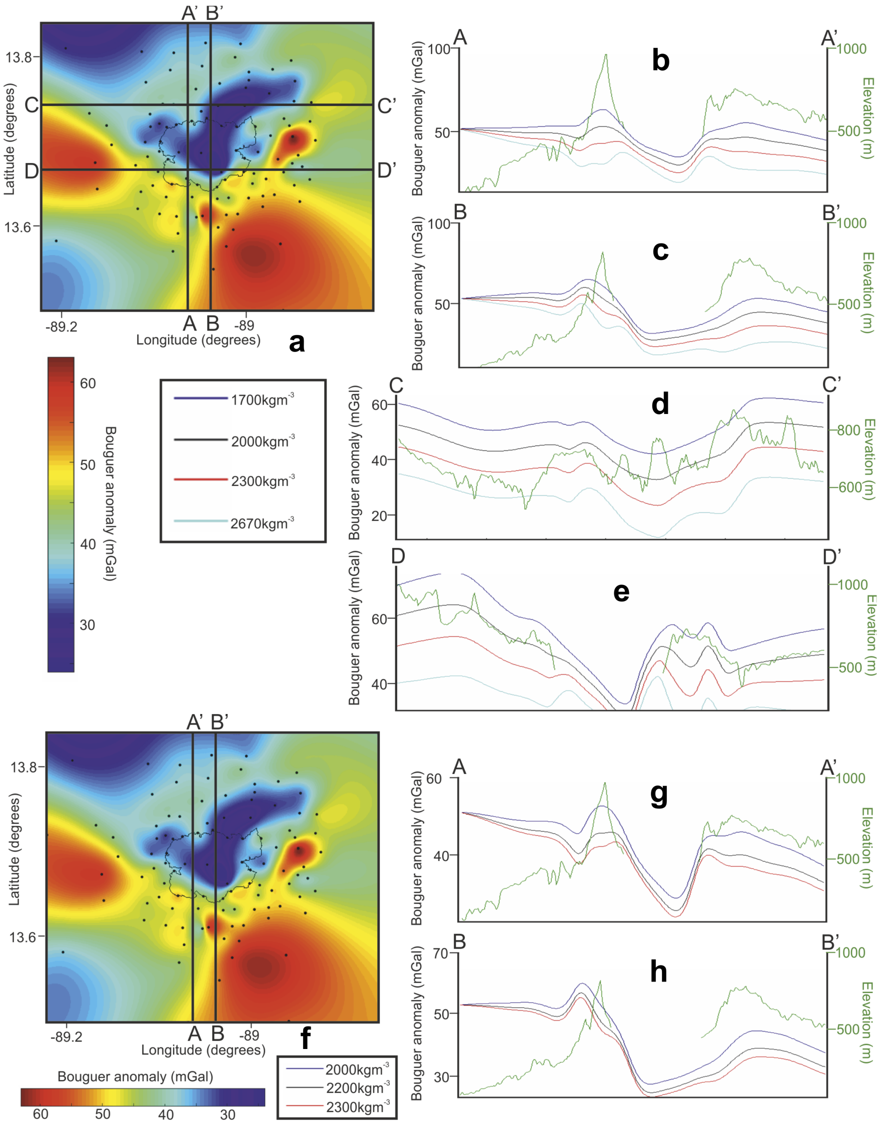

Supplementary Figure 3: Relationship between Bouguer anomaly and topography along several NS and EW profiles for different terrain densities between 1700 and 2670 kg m^{-3}. a) - e): Crude exploration of a possible correlation. f) - h): More refined exploration. The Bouguer anomaly resulting from the chosen terrain density of 2200 kg m^{-3}, which provides the least correlation (positive or negative) is also shown.

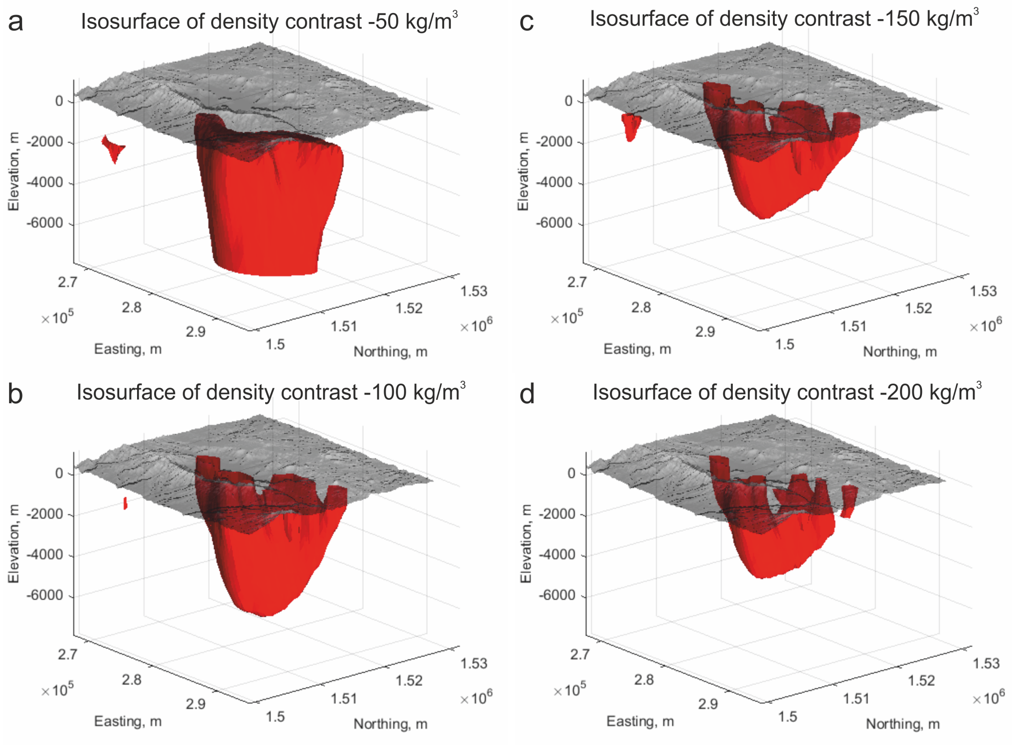

Supplementary Figure 4: Isosurface plots of negative density contrasts. Isosurfaces delineating a negative density contrast of half the prescribed minimum density contrast, for inversion runs using a stratified background with a linear density increase of 50 kg m^{-3} for each kilometre depth. a) Run using a priori minimum and maximum density contrasts of $\pm$100 kg m^{-3}, with a plotted isosurface showing a -50 kg m^{-3} contrast. b) Minimum and maximum $\pm$200 kg m^{-3}, plotted isosurface of -100 kg m^{-3}. c) Minimum and maximum $\pm$300 kg m^{-3}, plotted isosurface of -150 kg m^{-3}. d) Minimum and maximum $\pm$400 kg m^{-3}, plotted isosurface of -200 kg m^{-3}.

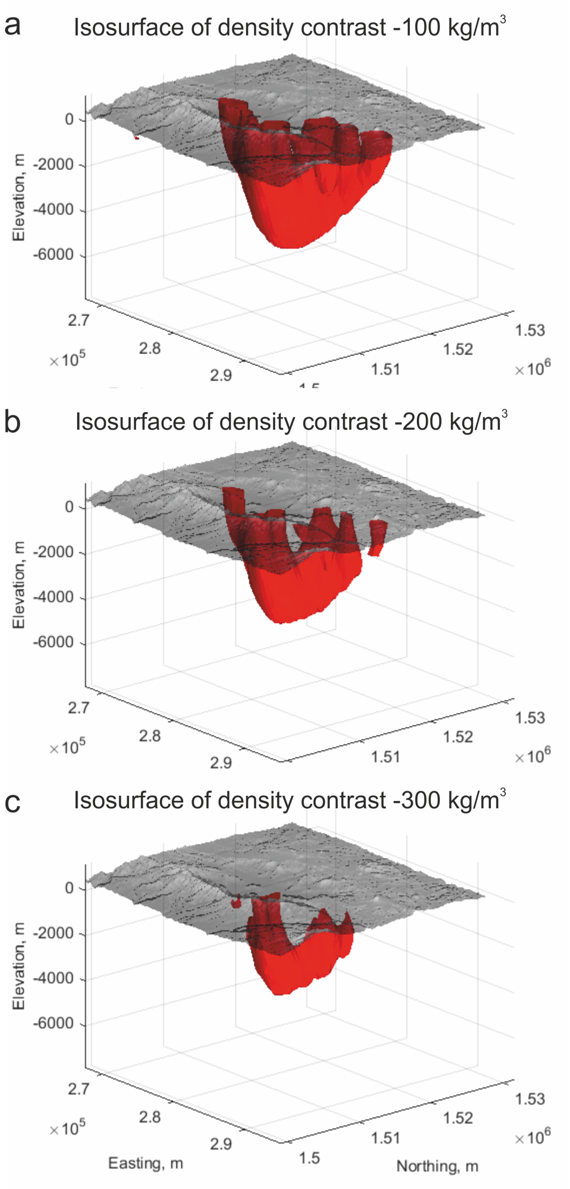

Supplementary Figure 5: Isosurfaces delineating the negative density contrasts of a) -100 kg m^{-3}, b) -200 kg m^{-3} and c) -300 kg m^{-3} for the inversion of a priori minimum and maximum density contrasts of $\pm$400 kg m^{-3} and a stratified background with a linear density increase of 50 kg m^{-3} for each kilometre depth.
